# Ganglion Cyst of the Foot Causing Second and Third Metatarsal Stress Reactions and Fractures: A Case Report

**DOI:** 10.7759/cureus.65387

**Published:** 2024-07-25

**Authors:** Yoshiharu Shimozono, Yasuyuki Mizuno, Noboru Funakoshi, Masahiko Kobayashi, Shuichi Matsuda, Fumiharu Yamashita

**Affiliations:** 1 Orthopaedic Surgery, Kyoto University Graduate School of Medicine, Kyoto, JPN; 2 Orthopaedic Surgery, Kyoto Shimogamo Hospital, Kyoto, JPN; 3 Orthopaedic Surgery, Kyoto University, Kyoto, JPN

**Keywords:** mri, metatarsal, stress fracture, foot, ganglion cyst

## Abstract

Ganglion cysts arising from the plantar aspect are rare, and the most common location of the foot and ankle is the dorsal aspect of the foot. We present a case of a 49-year-old man with pain in the right foot. Plain radiographs showed thinning of the cortical bone in the right second and third metatarsals, and MRI showed cystic lesions between the second and third metatarsals, resulting in stress reactions and fractures. The fracture gradually remodeled and completely healed following resection of the ganglion cysts. If plain radiographs show atypical changes in the metatarsal bone morphology, a ganglion cyst, as in this case, should be suspected, and MRI should be considered.

## Introduction

A ganglion is a benign cystic mass containing clear, high-viscosity mucinous fluid, encapsulated by dense fibrous connective tissue lined with flat spindle-shaped cells rich in hyaluronic acid and other mucopolysaccharides [1-3]. These cysts likely result from repetitive microtrauma, primarily in areas under constant mechanical stress, leading to mucinous degeneration of connective tissue. Ganglion cysts typically originate from the tendon sheath, muscles, nerves, or periosteum. They are most frequently found on the hand and wrist, followed by the ankle and foot [4,5]. While the dorsal aspect of the foot is the most common location for foot ganglions, their occurrence on the plantar aspect is rare [6]. Additionally, most patients with ganglion cysts on the foot report pain, often due to the compression of adjacent structures, such as superficial nerves [6]. We present a rare case involving a 49-year-old male who experienced foot pain associated with second and third metatarsal stress fractures/reactions with cortical thinning, attributed to a ganglion cyst arising from the plantar aspect of the foot.

## Case presentation

A 49-year-old male patient presented to the outpatient clinic with a two-month history of pain in his right foot, which had progressively worsened, impacting his ability to work and walk. Initially, he sought medical attention from a local doctor who, after conducting an X-ray, could not identify the cause of the pain. A steroid injection was administered to the dorsal foot without any improvement. Subsequently, the patient was referred to our clinic.

**Figure 1:**
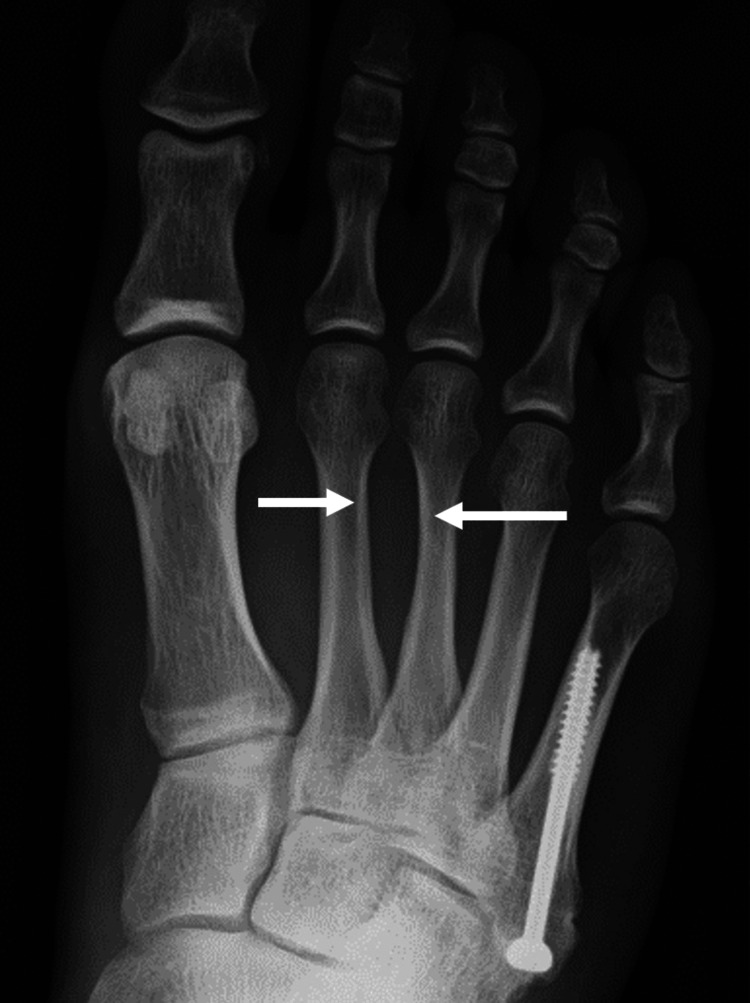
Plain radiograph showing cortical bone thinning in the right second and third metatarsals (white arrow).

**Figure 2:**
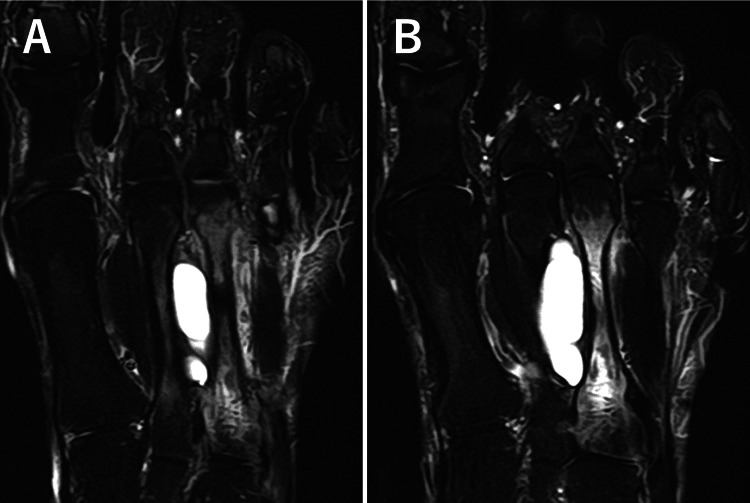
MRI of the right foot. T2-weighted image showing a high-intensity cystic mass lesion between the second and third metatarsals with high signal intensity in both metatarsals (A and B).

**Figure 3:**
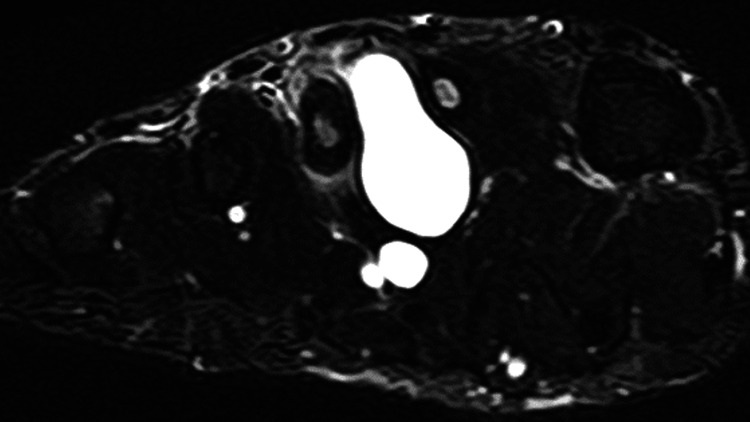
MRI axial image showing gourd-shaped cysts between the second and third metatarsals.

**Figure 4:**
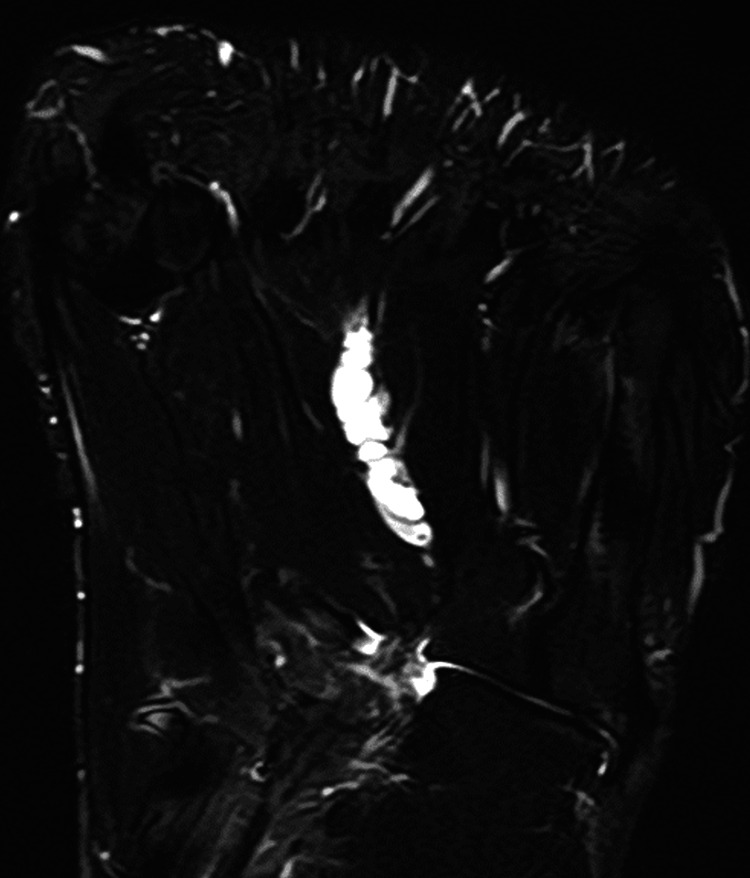
MRI of the right foot on the plantar aspect showing bead-like cysts.

**Figure 5:**
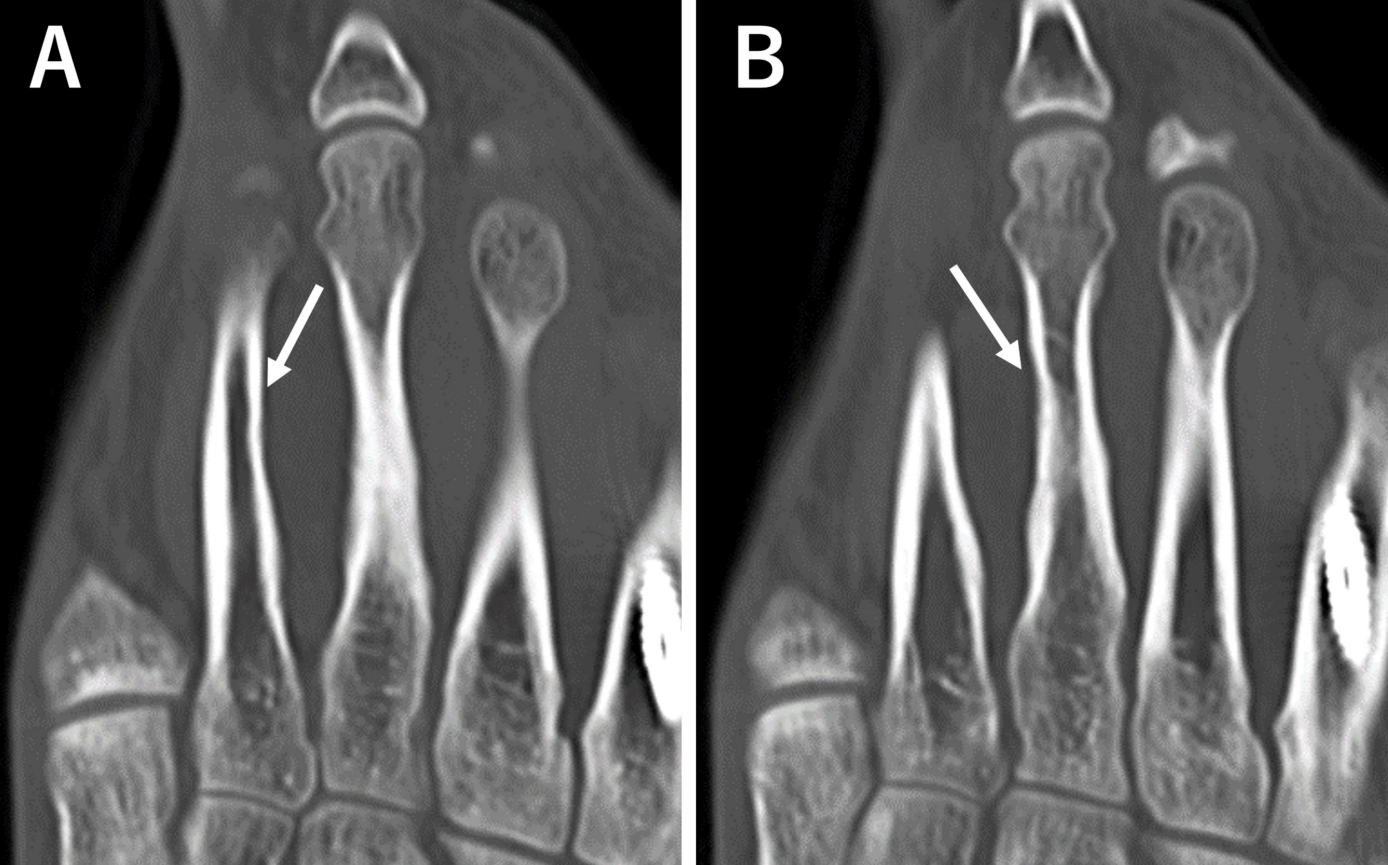
CT showing bone cortex thinning in the second (A) and third (B) metatarsals.

At the initial visit, physical examination revealed midfoot pain during loading and tenderness of the dorsal foot, with no overlying skin discoloration, redness, or punctum. Plain radiographs demonstrated cortical bone thinning in the right second and third metatarsals (Figure 1). Magnetic resonance imaging (MRI) revealed cystic lesions between the second and third metatarsals, characterized by low signal intensity on T1-weighted images and high signal intensity on T2-weighted images. Adjacent to the cystic lesion, the second and third metatarsals generally exhibited high signal intensities on T2-weighted images (Figure 2). High signal intensity in the third metatarsal extended throughout the entire metatarsal. MRI axial images showed gourd-shaped cysts between the second and third metatarsals, suggesting metatarsal compression (Figure 3). The cyst appeared to originate from the plantar side, though the exact origin was unclear (Figure 4). Computed tomography (CT) also showed cortex thinning in the second and third metatarsals (Figure 5). Based on these findings, the mass was diagnosed as a ganglion cyst adjacent to the metatarsals, causing pathologic second metatarsal stress reactions and third metatarsal stress fractures. The patient was offered conservative and surgical excision treatment options. As the pain continued to worsen, interfering with his daily activities and work, he opted for surgery. A ganglionectomy was performed.

**Figure 6:**
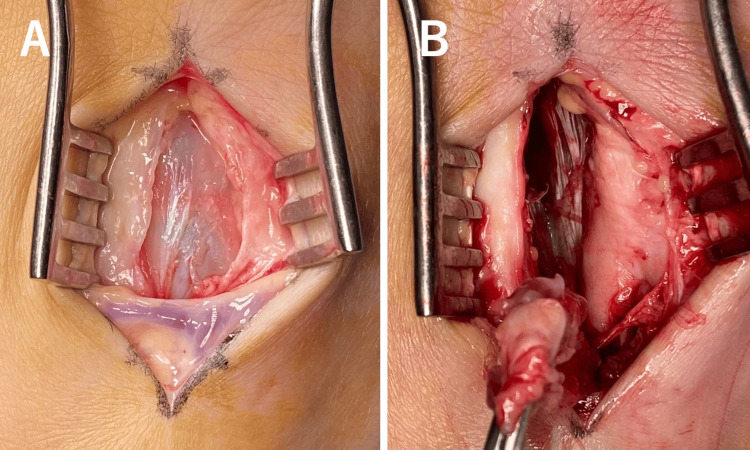
Dorsal view of the right foot showing a cystic lesion between the second and third metatarsals (A). The lesion was excised en bloc (B).

**Figure 7:**
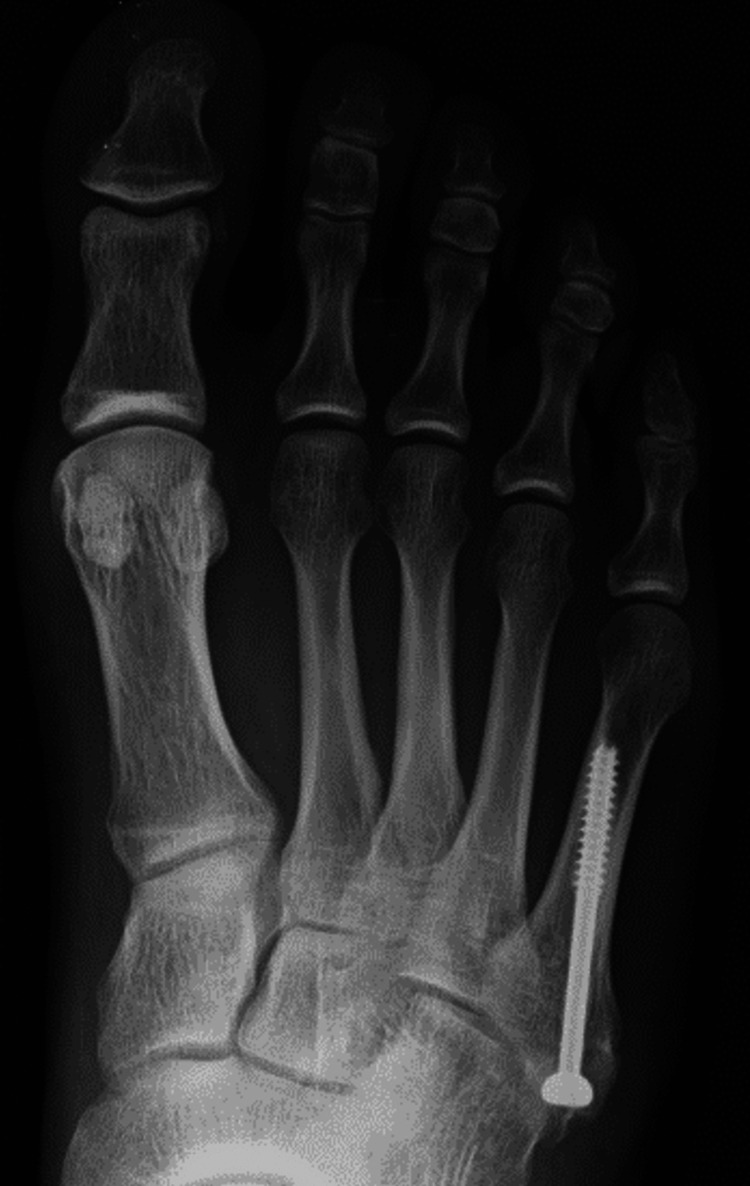
Plain radiograph at the 16-month follow-up visit showing healed and remodeled second and third metatarsals.

A longitudinal incision was made in the dorsal aspect of the foot between the second and third metatarsals to access the cystic lesion. The lesion was located and excised en bloc (Figure 6). The stem of the cyst was believed to originate from the flexor tendon sheath on the plantar side, although the exact source was difficult to determine. The cyst's compression caused stress fractures in the metatarsals. Histopathological examination revealed a mucus-containing cystic lesion. The cyst walls exhibited fibrous thickening, hyalinization, and mild mucinous degeneration. Epithelial cells were sporadically observed on the inner surface of the cysts. Therefore, the mass was diagnosed as a ganglion cyst. Postoperatively, the patient was allowed weight-bearing as tolerated. The wound healed without complications, and the patient's symptoms resolved completely. Postoperative remodeling of the fractures was gradual and completed 16 months after surgery (Figure 7). At the three-year follow-up, there was no recurrence of the ganglion or stress fractures.

## Discussion

In the foot and ankle, the most common location for ganglions is the dorsal aspect of the foot (62%), followed by the ankle (19%), with the plantar aspect being rare (2%) [6]. There are limited reports of stress fractures caused by ganglion cysts, and these are typically periosteal or intraosseous ganglions [7-9]. To the best of our knowledge, there have been no prior reports of metatarsal stress reactions or fractures resulting from pressure exerted by an extraosseous ganglion cyst, as observed in this case.

Wakabayashi et al. documented an intraosseous ganglion in the metatarsal where an osteolytic lesion was detected on plain radiographs [9]. Similarly, Serigano et al. reported a stress fracture of the third metatarsal caused by a periosteal ganglion [8]. However, the ganglion cyst in our case was significantly larger and had a stem extending from the plantar aspect, resulting in extraosseous compression of the second and third metatarsals. This is the first documented case of concurrent stress fractures in two metatarsals caused by compression from a ganglion cyst. Radiographic imaging typically reveals bony alterations, such as pressure absorption, sclerotic erosion of the underlying cortex, and periosteal reactions in cases of periosteal ganglion [10]. In the present case, plain radiographs showed an erosive cortical lesion in the metatarsal neck and diaphysis similarly, while MRI revealed both stress reactions/fractures and ganglion cysts.

Although the patient was not involved in sports, his physically demanding job as a landscaper likely contributed to the condition. Following the resection of the ganglion, the stress fracture sites underwent remodeling and fully recovered, enabling the patient to return to work pain-free, with no recurrence of symptoms observed.

## Conclusions

Ganglion cysts arising from the plantar aspect are exceedingly rare. This report presents a unique case where an enlarged ganglion compressed two metatarsals, resulting in stress reactions and fractures with cortical thinning. When plain radiographs show atypical changes in metatarsal bone morphology, a ganglion cyst should be suspected, and MRI should be considered for a definitive diagnosis. Surgical intervention can effectively treat such cysts, but it is essential to ensure thorough removal of the cyst from its stem to minimize the risk of recurrence.
